# *Rhus coriaria* induces senescence and autophagic cell death in breast cancer cells through a mechanism involving p38 and ERK1/2 activation

**DOI:** 10.1038/srep13013

**Published:** 2015-08-12

**Authors:** Hussain El Hasasna, Khawlah Athamneh, Halima Al Samri, Noushad Karuvantevida, Yusra Al Dhaheri, Soleiman Hisaindee, Gaber Ramadan, Nedaa Al Tamimi, Synan AbuQamar, Ali Eid, Rabah Iratni

**Affiliations:** 1 Department of Biology, College of Science, United Arab Emirates University, Al-Ain, P.O. Box 15551, United Arab Emirates; 2 Department of Chemistry, College of Science, United Arab Emirates University, Al-Ain, P.O. Box 15551, United Arab Emirates; 3 Department of Biological and Environmental Sciences, College of Arts and Sciences, Qatar University, P.O. Box: 2713, Doha, Qatar

## Abstract

Here, we investigated the anticancer effect of *Rhus coriaria* on three breast cancer cell lines. We demonstrated that *Rhus coriaria* ethanolic extract (RCE) inhibits the proliferation of these cell lines in a time- and concentration-dependent manner. RCE induced senescence and cell cycle arrest at G1 phase. These changes were concomitant with upregulation of p21, downregulation of cyclin D1, p27, PCNA, c-myc, phospho-RB and expression of senescence-associated β-galactosidase activity. No proliferative recovery was detected after RCE removal. Annexin V staining and PARP cleavage analysis revealed a minimal induction of apoptosis in MDA-MB-231 cells. Electron microscopy revealed the presence of autophagic vacuoles in RCE-treated cells. Interestingly, blocking autophagy by 3-methyladenine (3-MA) or chloroquine (CQ) reduced RCE-induced cell death and senescence. RCE was also found to activate p38 and ERK1/2 signaling pathways which coincided with induction of autophagy. Furthermore, we found that while both autophagy inhibitors abolished p38 phosphorylation, only CQ led to significant decrease in pERK1/2. Finally, RCE induced DNA damage and reduced mutant p53, two events that preceded autophagy. Our findings provide strong evidence that *R. coriaria* possesses strong anti-breast cancer activity through induction of senescence and autophagic cell death, making it a promising alternative or adjunct therapeutic candidate against breast cancer.

Breast cancer continues to be the second leading cause of cancer-related deaths in women. An approximate of 10 to 15% of breast cancer cases belong to the triple-negative breast cancer (TNBC) group of cancer. TNBC lack expression of estrogen, progesterone, and the HER-2 epidermal growth factor membrane receptors, are highly aggressive and invasive with poor prognosis of patients and, does not respond to hormonal therapies^1^. Currently, there is no defined standard treatment strategy for prevention of reoccurrence for this disease other than traditional chemotherapy.

Common cancer treatment drugs aim at inducing cell death, which is considered a prerequisite for preventing malignant cell growth. However, several studies demonstrated that cellular senescence, which also occurs *in vivo*, might provide a critical barrier for cancer development and progression^2^. In fact, a large body of evidence points to a crucial role for cellular senescence against tumorigenesis. Thus, the engagement of senescence may represent a key component for therapeutic intervention in the eradication of cancer^3^. Senescent cells are known to remain viable with sustained metabolism, but are in a state of stable growth arrest^4^. This arrest is achieved and maintained in either G1 or G2/M stages of cell cycle, at least through overexpression of specific cyclin-dependent kinase inhibitors such as p16, p21 and p27^5^,^6^,^7^,^8^. While p21 is important for cell cycle arrest that is associated with early senescence, p16 appears to be important for maintaining the senescence phenotype^5^. In fact, dephosphorylation of RB, which is considered a biochemical hallmark of senescence, seems to be induced by p21^9^. Moreover, p21 has been found to be upregulated in senescent cells and its forced overexpression is sufficient to induce cell cycle arrest and premature senescence in wild-type and mutant p53 cells such as MDA-MB-231 cells^10^.

Although apoptosis is a common mechanism for most of chemotherapeutics that induce cancer cell death, recently, the status of autophagy in cancer therapy has also been given increased attention. Autophagy is a highly conserved lysosomal degradation pathway by which misfolded or aggregated proteins, damaged organelles and intracellular pathogens are eliminated. Autophagy starts when such unnecessary byproducts and damaged organelles are engulfed into double-membrane vesicles (autophagosomes) and transported to lysosomes where autophagosomes fuse with lysosomes to form single-membrane autolysosomes and the inner engulfed materials are ultimately degraded and recycled^11^. As such, tight regulation of autophagic processes is integral to cellular homeostasis. Under normal conditions, basal autophagy functions to remove aged and damaged organelles and proteins. Excessive autophagy, however, may, lead to cell death referred to as type II programmed cell death (type II PCD)^12^.

Several anticancer drugs were shown to mediate their effect through DNA damage. In fact, several studies suggest that cells undergo premature senescence in response to DNA damaged by oxidative or genotoxic stressors^9^,^13^,^14^. Moreover, increasing evidence argues in favor of a role for DNA damage in the induction of autophagy; however, the mechanism through which DNA damage triggers autophagy and subsequent cellular responses remains unclear^15^,^16^,^17^.

Since prehistoric periods, plants have been used as medicine and cure for several diseases^18^. Most chemotherapeutic agents used today are linked to natural products. For example, more than half of all small-molecule new chemical entities introduced as drugs during the last three decades are intimately associated with natural products. Indeed, nearly three quarters of all cancer chemotherapeutic agents introduced since 1940 are either natural products or directly derived therefrom^19^. Identification and development of new chemotherapeutic agents from plants have gained significant recognition in the field of cancer therapy and became a major area of experimental cancer research.

Plant-derived anticancer drugs appear to not only be more effective than synthetic drugs, but they also have with less undesired side-effects. Examples of anticancer drugs derived from plants and are currently in clinical use include the *vinca* alkaloids vinblastine and vincristine that were isolated from *Catharan roseus*, the terpene paclitaxel from *Taxus brevifolia* Nutt., and the DNA topoisomerase I inhibitor camptothecin from *Camptotheca acuminata*^20^. Various natural products present in the diet were found to possess a wide variety of remarkable anti-breast cancer properties, such as (i) altering the effects of signaling pathways^21^,^22^ (ii) killing of breast cancer cells by modulating programmed cell death, primarily referring to apoptosis, and autophagy^23^ and (iii) prevent and/or suppress breast cancer progression in various preclinical animal models^24^.

*Rhus coriaria*. commonly known as sumac is a flowering shrub that belongs to Anacardiacea family. It is widely distributed in temperate and subtropical regions^25^ and its fruit forms clusters of reddish drupes^26^. In addition to its use as a culinary herb, *R. coriaria* is known to elicit many therapeutic values^27^,^28^. For example, *R. coriaria* possesses potent antioxidant activity due its phenolic compounds^11^,^29^. Several studies linked the accumulation of ROS (reactive oxygen species) in the body to different diseases such as atherosclerosis^30^, insulin resistance, type II diabetes^31^ cardiovascular diseases^18^, osteoarthritis^32^, hepatocytes toxicity^33^ and DNA damages^34^, where *R. coriaria* extract was found to have an effect on all of them. Moreover, *R. coriaria* extract reduces the postprandial blood glucose (PBG) in type II diabetic rats^35^. In addition, sumac possesses antimicrobial activity against Gram positive and Gram negative bacteria^36^. The phytochemical compounds of sumac have been characterized using HPLC–DAD–ESI-MS/MS method^37^. 211 phytochemicals were identified and these include organic acids, phenolic acids, phenolic compounds conjugated with malic acid derivatives, flavonoids, isoflavonoids, hydrolysable tannins, anthocyanins, terpenoids and other compounds (such as butein, Iridoid and coumarin derivatives). Interestingly, the anticancer potential of *Rhus coriaria* remains largely unexplored.

In the present study, we investigated the cytotoxic effects of *Rhus coriaria* extract against human breast cancer cells. Our results demonstrate that RCE exert its cytotoxic effect through the induction of growth inhibition, permanent cell cycle arrest, senescence, apoptotis and autophagic cell death in the highly metastatic triple negative MDA-MB-231 cells.

## Materials and Methods

### Cell culture, chemicals and antibodies

Human breast cancer cells MDA-MB-231 and MCF-7 were maintained in DMEM (Hyclone, Cramlington, UK) and T47D in RPMI (Hyclone, Cramlington, UK). All media were complemented with 10% fetal bovine serum (FBS) (Hyclone, Cramlington, UK) and 100 U/ml penicillin/streptomycin (Hyclone, Cramlington, UK). 3-methyadenine and chloroquine (CQ) were purchased from Millipore Analyzer (Millipore, Hayward, CA, USA) and Sigma-Aldrich (Saint-Quentin FAllavier, France), respectively. Antibodies to p62/SQSTMI and cleaved PARP were obtained from Abcam (Abcam, Cambridge, UK). Antibodies to LC3, p21 p27, and pErk1/2(Th202/Th204) and to Phospho-p38 MAPK (Thr180/Tyr182) were obtained from Cell Signaling (USA). Antibodies to γH2AX, p21 (WAFA/Cip1), p27 (Kip1), cyclin D1, PCNA, c-myc, Phospho-Rb (Ser807/Ser811) Beclin-1 and p53 were obtained from Millipore (Millipore, Hayward, CA, USA). Antibodies to p16 were obtained from BD Pharmingen (USA). Antibodies to β-actin were obtained from Santa Cruz Biotechnology, Inc (USA).

### Preparation of the *Rhus coriaria* Ethanolic Extract (RCE)

Fruits of *Rhus coriaria L.* were collected from a private farm located at 33° 16′ 35.59′′ N and 35° 19′ 02.89′′ E. The farm is located in Ma’rakeh, Tyre, Lebanon and the approval of the owner was obtained before collecting the fruit or commencing any experiments. This plant is neither endangered nor protected by any laws and it is readily and commercially available in the market. RCE was prepared as previously described^8^. Briefly 10.0 g of the dried fruit were ground to a fine powder using a porcelain mortar and pestle. The powder was then suspended in 100 mL of 70% absolute ethanol and the mixture was kept in the dark for 72 hours at 4 °C in a refrigerator without stirring. After that, the mixture was then filtered through a glass sintered funnel and the filtrate was evaporated to dryness using a rota-vapor at room temperature. The red residue was kept under vacuum for 2–3 hours and its mass was recorded. The residue was stored at −20 °C until further use.

### Measurement of cellular viability

Cells were seeded in triplicate in 96-well plates at a density of 5,000 cells/well. After 24 h of culture, cells were treated with or without various concentrations of *Rhus coriaria* extract for different durations. Cell viability was measured with the Cell Cytotoxicity Assay Kit (Abcam) according to the manufacturer’s specifications. The results are representative of an average of 5 independent experiments. Data were presented as proportional viability (%) by comparing the treated group with the untreated cells, the viability of which is assumed to be 100%.

Cell viability was also measured using the Muse™ Cell Analyzer (Millipore, Hayward, CA, USA) using the Muse Count and Viability Kit (Millipore, Hayward, CA, USA) which differentially stains viable and dead cells based on their permeability to two DNA binding dyes. Briefly, cells were plated onto 12-well plates (50 × 10^4^ cells/well) and allowed to grow for 24 h. The day of treatment cells were counted to estimate the approximate number of cells per well. Following RCE treatment at indicated times, viable cells were counted using Muse™ Cell Analyzer.

### Cell cycle analysis

The cell cycle distribution analysis in control and RCE-treated MDA-MB-231 cells was performed with the Muse™ Cell Analyzer (Millipore, Hayward, CA, USA) using the Muse™ Cell Cycle Kit (Millipore, Hayward, CA, USA) according to the manufacturer’s instructions. Briefly, cells grown onto 6 cm culture dishes were treated with or without various concentrations of RCE. After 24 h or 48 h of treatment, cells were collected by trypsinization, washed in PBS and resuspended in complete media and the Muse cell cycle test reagent was then added to each test tube. Cells were then incubated for 30 min at room temperature in the dark. After staining, the cells were processed for cell cycle analysis. Percentage of cells in G0/G1, S and G2/M phases were determined using the FlowJo software.

### Quantification of apoptosis by Annexin V labelling

Apoptosis was examined using the Annexin V & Dead Cell kit (Millipore, Hayward, CA, USA) according to the manufacturer’s instructions. Briefly, MDA-MB-231 cells were treated with or without RCE for 48 h. Detached and adherent cells were collected and incubated with Annexin V and 7-AAD, a dead cell marker, for 20 min at room temperature in the dark. The events for live, early and late apoptotic cells were counted with the Muse™ Cell Analyzer (Millipore, Hayward, CA, USA).

### Transmission electron microscopy (TEM)

TEM was carried out as previously described^38^. Briefly, control and RCE-treated cells were fixed overnight at 4 °C in 2% paraformaldehyde, 2.5% glutaraldehyde in 0.1M sodium cacodylate buffer pH 7.2 overnight at 4 °C, before being post-fixed with 1% OsO_4_ for 1 h. Cells were then dehydrated in a graded ethanol series and embedded in Agar 100 epoxy resin. Ultrathin sections were mounted on Cu grids and stained first with uranyl acetate followed by lead citrate. Sections were observed and photographed under a Philips CM10 Transmission Electron Microscope.

### Hematoxylin-eosin staining of cells

MDA-MB 231 cells (5 × 10^4^) were grown on 2 well labtek chamber slide for 24 h, then treated with and without RCE for 48 h. Cells were then washed twice with PBS and fixed in 10% formalin solution (4% paraformaldehyde) for 5 min at room temperature followed by permeabilization in 70% ethanol. Cells were then washed three times with PBS, stained with hematoxylin for 1 min and washed again before staining with eosin for 30 seconds. For viewing the cells, slides were mounted with 50% glycerol, sealed and observed under Olympus microscope (BX41) fitted with Olympus camera (DP71).

### Senescence-Associated-β-Galactosidase (SA-β-gal) staining

Briefly 10^5^ MDA-MB-231 cells were cultured in 6 well plate and treated with and without RCE for 48 h. Treated and control cells were then washed in PBS, and fixed with 2% formaldehyde/0.2% glutaraldehyde for 5 min at room temperature. The SA-β-gal staining was performed as previously described^39^.

### Whole Cell extract and Western Blotting analysis

Cells (1.8 × 10^6^) were seeded in 10 cm culture dishes and cultured for 24 h before addition of RCE. After incubation, cells were washed twice with ice-cold PBS, scraped, pelleted and lysed in RIPA buffer (Pierce) supplemented with protease inhibitor cocktail (Roche) and phosphatase inhibitor (Roche). After incubation for 30 min on ice, cell lysates were centrifuged at 14,000 rpm for 20 min at 4 °C. Protein concentration of lysates was determined by BCA protein assay kit (Thermo Scientific) and the lysates were adjusted with lysis buffer. Aliquots of 25 μg of total cell lysate were resolved onto 8–15% SDS-PAGE. Proteins were transferred to nitrocellulose membranes (Thermo Scientific) and blocked for 1 h at room temperature with 5% non-fat dry milk in TBST (Tris-buffered saline with 0.05% Tween 20). Incubation with specific primary antibodies was performed in blocking buffer overnight at 4 °C. Horseradish peroxidase-conjugated anti-IgG was used as secondary antibody. Immunoreactive bands were detected by ECL chemiluminescent substrate (Thermo Scientific). Where needed, membranes were stripped in Restore western blot stripping buffer (Thermo Scientific) according to the manufacturer’s instructions.

### Statistical analysis

The statistical analysis were done using SPSS version 21. Data were reported as group mean ± SEM. The data were analyzed via t-test, univariate test and one-way ANOVA followed by LSD’s Post-Hoc multiple comparison test (to compare all groups). Significance for all statistical comparisons was set at p < 0.05 using a two-tailed test.

## Results

### Growth inhibitory effect of *Rhus coriaria* extract on MDA-MB-231, MCF-7 and T47D breast cancer cells

**Figure 1:**
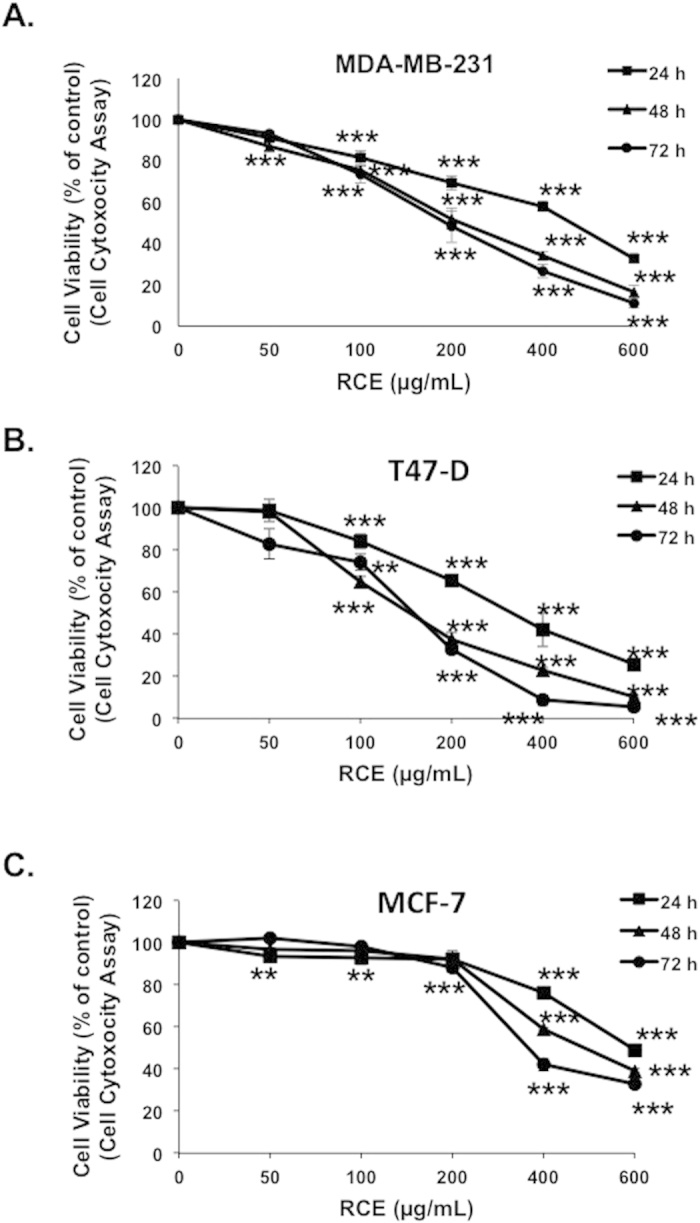
Inhibition of cellular viability by *Rhus coriaria.* Exponentially growing MDA-MB-231 (**A**), T47-D cells (**B**), and MCF7 (**C**) breast cancer cells were treated with and without the indicated concentrations of RCE for 24, 48 and 72 h. Viability monitored as described in Materials and Methods. Data represent the mean of five independent experiments carried out in triplicate. Statistical analysis for cell viability data was performed using using one-way ANOVA followed by LSD Post-Hoc test (*p < *0.05*, **p < *0.005*, ***p < *0.001*).

To examine the anticancer activity of *Rhus coriaria* extract (RCE) on breast cancer cells, we first measured the effect of various concentrations of the extract (0, 50, 100, 200, 400 and 600 μg/mL) on the proliferation of three different (MDA-MB-231, MCF-7 and T47D) breast cancer cell lines (Fig. 1A–C). Our results show that exposure of the three breast cancer cell lines to RCE decreased cellular viability in concentration and time-dependent manners. Based on the determined IC_50_ for each cell line (Supplementary Table 1), it appears that T47D and MDA-MB-231 cells exhibit a greater sensitivity to RCE compared to the MCF-7 cells. We next focused on the triple negative and highly metastatic breast cancer MDA-MB-231 cells to investigate the mechanism by which RCE decreased cell viability of breast cancer cells. In order to distinguish between cell death and possible growth arrest effects of RCE, cell viability of the MDA-MB-231 cells in response to RCE treatment, was monitored by counting the viable cells using the Muse cell analyzer (Millipore) as described in the material and methods section. We found that RCE treatment also led to a time- and concentration-dependent decline in the number of viable cells, indicative of cell death, when compared to the number of cells counted at the day of treatment (day 0) (Supplementary Figure 1).

### *Rhus coriaria* induces morphological changes in MDA-MB-231 cells

**Figure 2:**
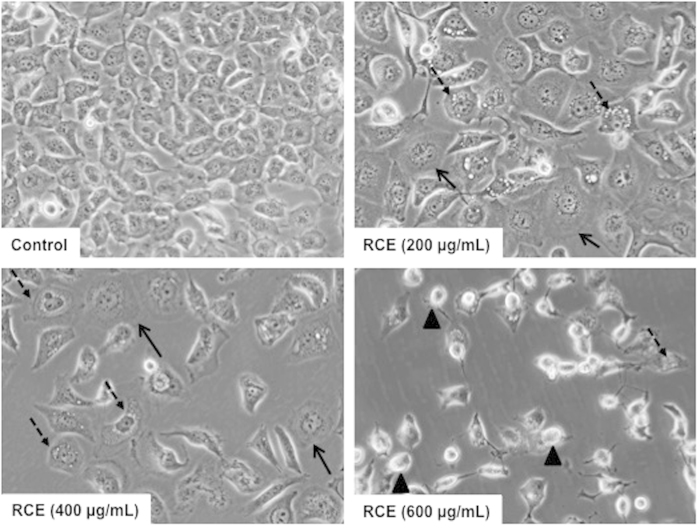
Morphological changes in RCE-treated MDA-MB 231 cells. Morphological changes observed in the MDA-MB 231 cells after 48 h of treatment with various concentration of RCE. Cells were observed under EVOS XL Core Cell Imaging System (Life Technologies).

Light microscopy observation of RCE-treated MDA-MB-231 cells revealed several morphological changes induced by this extract. As shown in Fig. 2, MDA-MB-231 cells treated with various concentrations of RCE (200, 400 and 600 μg/mL), underwent morphological changes visible after 24 h of treatment. A subpopulation of treated cells exhibited a senescence-like phenotype characterized by cell size increase and flattening shape compared to control cells (Fig. 2, plain arrows). Moreover, treated cells also revealed cytoplasmic vacuolation (Fig. 2, dashed arrows). At higher concentrations of RCE (600 μg/mL), cells appeared smaller and rounded, characteristic of dying cells (Fig. 2, arrow heads).

### *Rhus coriaria* extract induces G1 arrest and senescence in breast cancer cells

**Figure 3:**
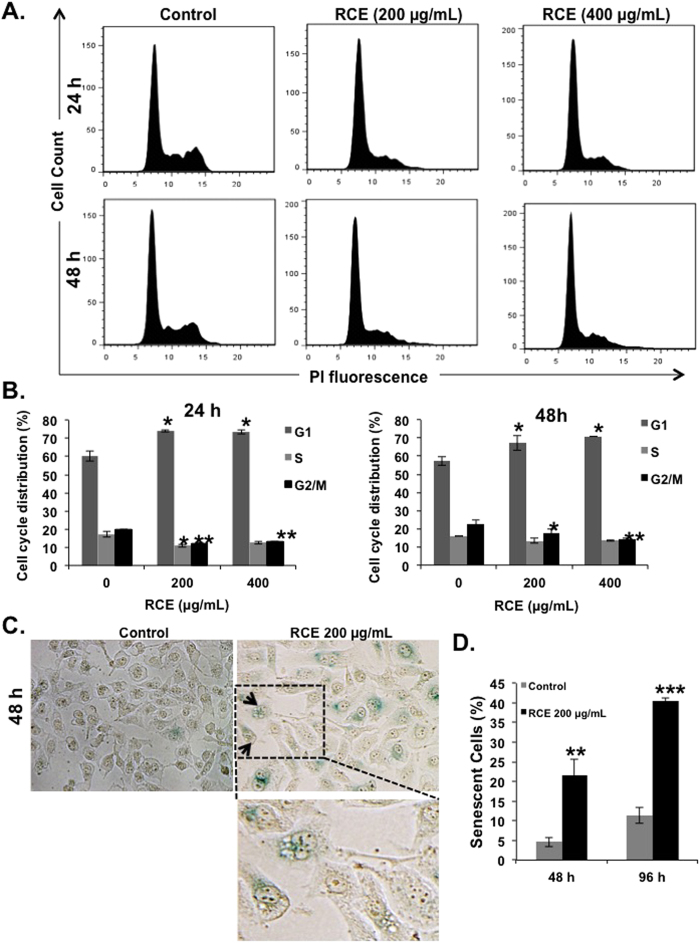
*Rhus coriaria* treatment induces G1 arrest and senescence in MDA-MB-231 cells. (**A–B**) Cell cycle distribution analysis of RCE-induced G1 cell-cycle block. MDA-MB 231 cells were first treated with RCE at the indicated concentrations for 24 h and 48 h, and then analyzed with Muse TM Cell Analyzer as described in Materials and Methods. (**C**–**D**) MDA-MB 231 cells were incubated with RCE (200 μg/mL) for 48 and 96 hours and stained for SA-β-Galactosidase activity to detect senescence. Data are representative of three independent experiments. Statistical analysis was performed using one-way ANOVA (*p < *0.05*, **p < *0.005*, ***p < *0.001*).

To investigate the mechanism(s) underlying the RCE inhibitory activity on breast cancer cells, we first examined its effect on cell cycle progression. Toward this, MDA-MB-231 cells were treated with indicated concentrations of RCE for 24 and 48 h and subjected to cell cycle analysis. We found that treating the cells with RCE caused significant inhibition of cell cycle progression in MDA-MB-231 cells at 24 and 48 h leading to an increase in the G1 population. In fact, the G1 population rose from 57% ± 2 in control cells to 67% ± 4 and 71% ± 0.1 in cells treated for 48 h with 200 and 400 μg/mL RCE (Fig. 3A,B), respectively.

The light microscopy observations noted in Fig. 2 along with the G1 arrest, often implicated in senescence, prompted us to examine whether the cell cycle arrested MDA-MB-231 cells did indeed undergo senescence. It is well known that senescent cells express a senescence-associated β-galactosidase (SA-β-Gal). Our results show that after treating the cells for 48 h, senescence became significantly detectable. After 48 h of treating cells with 200 μg/mL RCE, 21% of cells expressed SA-β-galctosidase (Fig. 3C,D); this proportion of senescent cells nearly doubled after 96 h of treatment. Taken together, these data suggest that induction of senescence might contribute to the inhibitory effect of RCE on the proliferation of MDA-MB-231 cells.

### Alteration of selected proteins associated with G1 block and senescence, and absence of proliferative recovery in RCE-treated MDA-MB-231 cells

**Figure 4:**
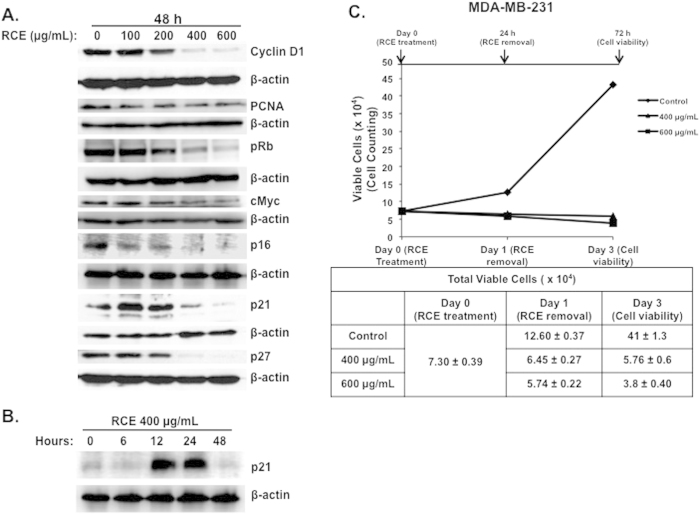
*Rhus coriaria* alters the expression of cell cycle regulators and inhibits proliferative recovery of breast cancer cells. (**A**) Alteration in proteins associated with cell cycle regulation and senescence in MDA-MB-231 cells, in response to *Rhus coriaria*. Cells were treated with or without the indicated concentrations of RCE for 48 h, and the protein level of Cyclin D1, PCNA, pRB, c-myc, p16, p21 and p27 was examined by western blotting. (**B**) Time-course accumulation of p21 in treated MDA-MB-231 cells. Cells were treated with 400 μg/mL RCE and a protein level of p21 was determined by western blot at different time-point (6, 12, 24 and 48 h) post-treatment. (**C**) Inhibition of cell viability recovery after RCE removal. MDA-MB-231 cells were exposed to RCE for 24 h, then, cells were washed to remove RCE and allowed to grow for another 48 h in fresh complete media. Cell viability was monitored using the Muse Cell Analyzer as described in material and methods. Data represent the mean ± SEM of three independent experiments.

Next, we evaluated the effect of RCE on a battery of proteins that are known to regulate cellular proliferation. Overexpression of cyclin D1 has been reported to shorten the G1 phase and occurs in many types of human cancer, whereas inhibition of its expression blocks G1-S transition^40^. As it is shown in Fig. 4A, the protein level of cyclin D1 decreased upon treatment with RCE in time- and concentration-dependent manners. Remarkably, cyclin D1 was almost undetectable with high concentrations of RCE (400 and 600 μg/mL). The expression of PCNA, an auxiliary protein involved in the control of eukaryotic DNA replication and required for cell cycle progression from G1 to S phase^41^, was also downregulated in response to RCE (Fig. 4A). Further, we checked for the expression of the proto-oncogene c-myc, a protein reported to regulate a myriad of target genes involved in cell proliferation, apoptosis and metabolism^42^ and whose inactivation has been reported to promote tumor regression by inducing senescence^43^. Figure 4A also shows that the level of c-myc protein was markedly downregulated in MDA-MB-231 cells in response to RCE treatment. The RB tumor suppressor protein is a cell cycle regulator, where hypophosphorylated RB is associated with G1 arrest and its phosphorylation in G1 allows progression from G1 to S^44^. We found that the level phosphorylated RB (pRB) dramatically decreased in concentration-dependent manner in RCE-treated cells (Fig. 4A). Because p21, a CDK inhibitor, protein has been reported to cause blockade of the G1-S transition, inhibit apoptosis and mediate senescence^45^,^46^, we investigated whether the growth inhibition and senescence mediated by RCE was associated with an induction of p21. The results of a typical experiment shown in Fig. 4A, demonstrates a significant increase of p21 protein only at a concentration of 100 and 200 μg/mL. Surprisingly, the protein level of p21 was lower in treated cells compared to untreated cells when higher concentrations of RCE (400 and 600 μg/mL) were used. The absence of p21 upregulation at 48 h post-treatment in cells treated with high concentration of RCE was intriguing, because G1 arrest as well as massive autophagy were also observed under these conditions in MDA-MB-231 cells. This observation prompted us to measure the level of p21 in cells treated with 400 at different time point post-treatment. Interestingly, we found a robust increase in the p21 level starting at 6 h and continued for 24 h, than dramatically decreased at 48 h (Fig. 4B). The level of two other CDK inhibitors, p16 and p27, was also examined in RCE-treated cells and was found to decrease significantly in concentration-dependent manner (Fig. 4A).

Next, we examined whether or not, in addition to its ability to inhibit the proliferation and to induce cell death, RCE can also suppress the potential of breast cancer cells to recover proliferative capability. Toward this, cells were first, treated with the indicated concentration of RCE for 24 h, and then cells were washed with PBS and placed in fresh complete media in the absence of RCE, and allowed to grow for another 48 h before assessing cell viability by cell counting. Figure 4C, shows that MDA-MB-231 cells failed to show proliferative recovery as the number of viable cells kept reducing even after RCE removal. Thus, our result indicates that RCE exerts an irreversible antiproliferative effect on cancer cells.

### Minimal induction of apoptosis by *Rhus coriaria* extract in MDA-MB-231 cells

**Figure 5:**
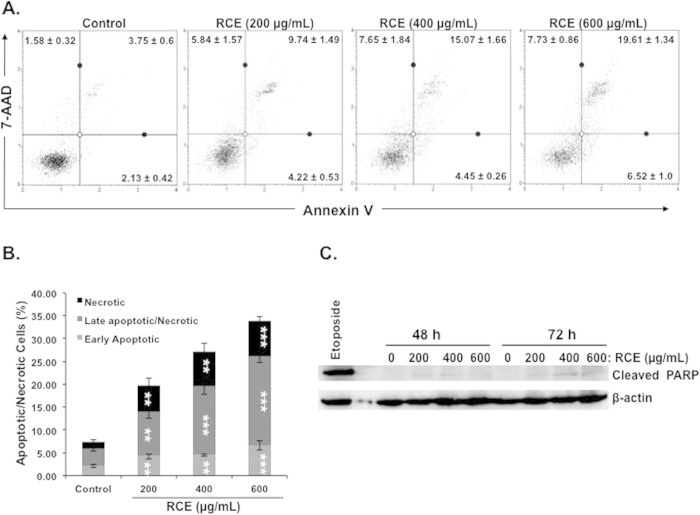
Determination of apoptosis in *Rhus coriaria*-treated cells. (**A–B**) Minimal induction of apoptosis in the MDA-MB-231 cells. Annexin V binding was carried out using Annexin V & Dead Cell kit (Millipore). Cells were treated with or without increasing concentrations of RCE for 48 h. Detached and adherent cells were collected and stained with Annexin V and 7-AAD and then the events for early and late apoptotic cells were counted with the Muse Cell Analyzer as described in Materials and Methods. Data represent the mean ± SEM of at least 3 independent experiments. Statistical analysis was performed using ANOVA followed by LSD Post-Hoc test to determine the significance (*p < *0.05*, **p < *0.005*, ***p < *0.001*). (**C**) Western blot analysis of PARP cleavage in MDA-MB-231. Cells were treated with increasing concentrations of RCE (200, 400 and 600 μg/mL) for 48 h and 72 h. Exposure of cell to etoposide (50 μM) for 24 h was used as a positive control for apoptosis. The western blots shown are representative of three independent experiments.

Because both cytotoxicity (Fig. 1A) and cell counting assay (supplementary Figure 1), showed that RCE induced cell death in MDA-MB-231 cells, we decided to investigate whether this is associated with induction of apoptosis. Annexin V staining was used to determine the percentage of apoptotic cells induced by RCE after 48 h treatment. As it is shown in Fig. 5A,B, exposure to RCE did not lead to a significant change (~6%) in the early stage apoptotic population (Annexin V^+^/7-AAD^−^). An increase but still minimal (<20%) in the late stage apoptotic/necrotic cells (Annexin V^+^/PI^+^)/was observed at highest RCE concentration (Fig. 5A,B), suggesting minimal apoptotic cell death induced by RCE in MDA-MB-231 cells.

Apoptosis was further assessed by PARP cleavage. Cells treated with etoposide (50 μM) for 24 h, a condition that was reported to induce apoptosis, was used as positive control. Despite the high level of concentration- and time-dependent cell death observed by cell toxicity and cell counting assay during the first 72 h of RCE treatment, very little PARP cleavage, indicative of apoptosis, was observed in RCE-treated MDA-MB-231 cells (Fig. 5C). Altogether, Annexin V staining and PARP cleavage data support the conclusion of minimal induction of apoptosis by RCE in MDA-MB-231 cells.

### Induction of autophagy in breast cancer cells by *Rhus coriaria*

**Figure 6:**
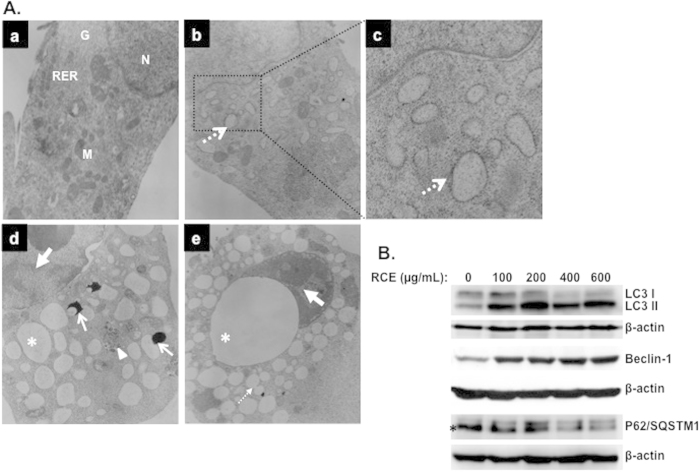
*Rhus coriaria* induces autophagy in MDA-MB-231 cells. (**A**) Representative electron micrographs of untreated MDA-MB-231 cells (a) and MDA-MB-231 cells treated with 400 μM RCE for 48 h (b–e). (**B**) Western blotting analysis of LC3II, p62(SQSTM1), and Beclin-1 expression RCE-treated MD-MB 231 cells. Cells were treated with or without increasing concentration of RCE for 48 h, then whole cell proteins were extracted and subjected to Western blot analysis, as described in materials and methods, for LC3II, 62(SQSTM1), Beclin1 and β-actin (loading control) proteins. The western blots shown are representative of at least three independent experiments.

Light microscopy observation (Fig. 2, dashed arrows) and eosin/hematoxylin staining (Supplementary Figure 2, plain arrows) of MDA-MB-231 cells treated with RCE revealed massive cytoplasmic vacuolation that might indicate induction of autophagy. In order to determine whether indeed this vacuolation resulted from activation of autophagy, we examined by transmission electron microscopy, the internal ultrastructure of MDA-MB-231 cells treated for 48 h with and without RCE. As it appears in Fig. 6A (panel a), control MDA-MB-231 cells showed an intact nuclei (N) and well developed organelles such as endoplasmic reticulum (RER), Golgi (G) and numerous mitochondria (M). In REC-treated MDA-MB-231 cells, a clearly visible double-membrane surrounded autophagosomes engulfing many cellular organelles can be seen (panel d, arrowhead). Interestingly, large number of swollen Rough Endoplasmic Reticulum (RER) that were studded with ribosomes could be clearly identified (panel b and c, dashed arrows) suggestive of ER stress in RCE-treated cells. Moreover, Autophagolysosomes at different stage of formation by fusion of organelles such as RER and lysosomes were also observed (panel d, plain arrows). Such fusion results in the formation of an increasing number of empty vacuoles, which then fuses (panel e, thin dashed arrow) to form very large number of macro-vacuoles that occupy most of the cytoplasmic space (panel e, asterisk). In some cells, very few organelles were present probably due to massive degradation through autophagolysosome. Moreover, chromatin condensation and damaged nuclear envelope (panel d and e, bold arrow) were also obvious in cells undergoing autophagy.

To further confirm autophagy induction in RCE-treated MDA-MB-231 cells, LC3II accumulation was analyzed by Western blotting in MDA-MB-231 treated with various concentration of RCE. Autophagy is characterized by the conversion of LC3I (cytosolic form) into a lipidized LC3II (autophagosome membrane-bound form). As it is shown in Fig. 6B, RCE induced a concentration-dependent accumulation of the LC3-II. The expression of another widely used autophagy-specific marker p62(SQSTM1), a ubiquitin-binding protein involved in autophagy and whose level decreases when autophagy flux increases, was also evaluated. Figure 6B, shows a concentration-dependent decrease in p62(SQSTM1). Hence, the conversion of LC3I/II along with the downregulation of p62 (SQSTM1) confirm the formation of autophagosome in RCE-treated MDA-MB-231 cells. Next we assessed the expression of Beclin-1, an important autophagy effector that plays a key role in autophagosome formation and whose expression is upregulated during autophagy induction. Western blotting data showed that the level of Beclin-1 also increased in concentration-dependent manner. Taken together, western blotting results along with electron microscopy observations, confirms the activation of autophagy in breast cancer cells in response to RCE treatment. Because, senescence become obvious only after 48 h treatment while autophagy was detectable as early as 12 h post-treatment, it is clear that autophagy is an early event that precedes senescence.

### Blockade of autophagy reduces cell death and senescence in *Rhus coriaria*-treated cells

**Figure 7:**
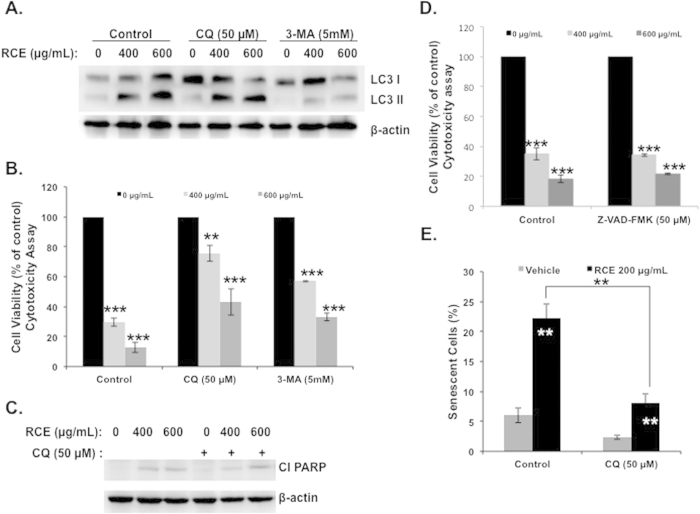
Effects of autophagy inhibitors on cell death, apoptosis and senescence. (**A**) Analysis of LC3-II accumulation in MDA-MB-231 cells. Cells were pretreated with or without 3-MA (5 mM) or CQ (50 μM) for 1 h and then RCE was added at the indicated concentrations for 48 h. Proteins were extracted and LC3-II accumulation was determined by western blot. (**B**) Inhibition of autophagy reduces cell death induced by RCE. MDA-MB-231 cells were pretreated as described above and treated for 48 h with 400 or 600 μg/mL RCE. Cell viability was determined using cytotoxicity assay kit as described in material and methods. Data are representative of three independent experiments. Statistical analysis of cell viability on control or treated cellswas performed using one-way ANOVA followed by LSD Post-Hoc test to determine significance (*p < *0.05*, **p < *0.005*, ***p < *0.001*). (**C**) Western blot quantification of cleaved PARP in cells pretreated with and without autophagy inhibitors. (**D**) Effect of caspase inhibition of cell viability of RCE-treated MDA-MB-231 cells. MDA-MB-231 cells were pretreated with the pan-caspase inhibitor Z-VAD-FMK (50 μM) for 1 h and then treated for 48 h with 400 or 600 μg/mL RCE. Cell viability was determined using cytotoxicity assay kit as described in material and methods. Data are representative of three independent experiments. Statistical analysis of cell viability on control or treated cellswas performed using one-way ANOVA followed by LSD Post-Hoc test to determine significance (*p < *0.05*, **p < *0.005*, ***p < *0.001*). (**E**) Effect of autophagy blockade on RCE-induced senescence. Cells were treated as described in A and stained, as described in material and methods, for SA-β-Gal activity to detect senescence. Data are representative of three independent experiments. Statistical analysis of senescent cells count on control or treated cells was performed using one-way ANOVA and univariate test to determine significance (**p < 0.01).

The observation that RCE induces robust cell death in MDA-MB-231 cells and, that induction of apoptosis is minimal raised the question of whether autophagy is responsible for the cytotoxicity activity of RCE through activation of type II programmed cell death and therefore its blockade by autophagy inhibitors might render cells less susceptible to RCE treatment. We used two widely used autophagy inhibitors, 3-Methyladenine (3-MA), which blocks autophagosome formation and chloroquine (CQ) which inhibits autophagosomal degradation. Results showed that autophagy was markedly inhibited by 3-MA, evident by decreased conversion of LC3-I to LC3-II (Fig. 7A). However, when cells were pre-treated with CQ, LC3-II protein accumulated to some extent (Fig. 7A). We found that when MDA-MB-231 cells were treated with RCE in the presence of 3-MA or CQ, cell viability was markedly improved when compared with RCE alone (Fig. 7B), suggesting that RCE-induced cell death is significantly dependent on autophagy induction. Furthermore, inhibition of autophagy neither increased nor reduced the level of cleaved PARP (Fig. 7C), suggesting that RCE-mediated apoptosis and autophagy in MDA-MB-231 might occur independently from each other. To further confirm that autophagy is the main mechanism of cell death in response to RCE, we decided to block apoptosis activation by inhibiting the activity of caspases. Toward this, we used the pancaspase inhibitor Z-VAD-FMK (50 μM). As it is shown in Fig. 7D, inhibition of apoptosis did not lead to significant recovery in cellular proliferation. This result was a bit surprising because apoptosis was also activated, although at minimal level, in RCE-treated cells. Interestingly, several studies reported that blockade of caspases activation, by caspase inhibitor, lead to autophagic cell death^47^,^48^. Hence, we hypothesize, that by blocking apoptosis in RCE-treated cells, the cells switches to the activation of autophagic cell death program. Altogether, our results demonstrate that autophagy is the main mechanism of cell death of breast cancer cells in response to RCE.

Because blockade of autophagy improved cell viability (Fig. 7B), and because SA-β-galactosidase was also detected in autophagic cells (Fig. 3C, small arrows), we asked the question whether blocking autophagy can also affect senescence in RCE-treated cells. Figure 7D shows that the number of senescent cells in wells containing both CQ and RCE is significantly lower than that in well treated with RCE alone. These results suggest that autophagy and senescence are linked events and that induction of senescence is at least partly dependent upon the activation of autophagy.

### Possible involvement of 38 and ERK signaling pathways in the induction of autophagy by *Rhus coriaria* in MDA-MB-231 cells

**Figure 8:**
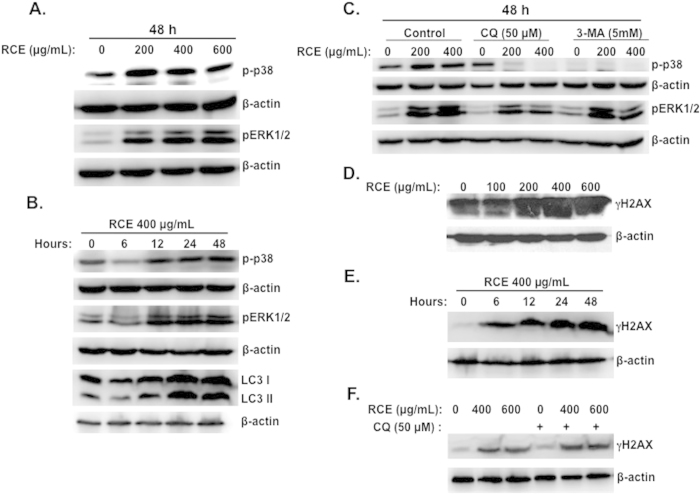
Activation of p38 and ERK1/2 and accumulation of DNA damage in RCE-treated MDA-MB-231 cells. (**A**) Concentration-dependent accumulation of phospho-p38 and pERK1/2in RCE-treated cells. MDA-MB-231 cells were treated with and without increasing concentrations of RCE for 48 h and p-p38 and ERK1/2 levels were determined by western blot. (**B**) Time-course accumulation of p-p38 and pERK1/2 in treated MDA-MB-231 cells. Cells were treated with 400 μg/mL RCE and protein levels of p-p38 and pERK1/2 was determined by western blot at different time-point (6, 12, 24 and 48 h) post-treatment. (**C**) Effects of autophagy inhibitors on the activation of p-p38 and pERK1/2. Cells were pretreated with or without 3-MA (5 mM) or CQ (50 μM) for 1 h and then RCE was added at the indicated concentrations for 48 h. Proteins were extracted and levels of p-p38 and pERK1/2 was determined by western blot. (**D**) Concentration-dependent accumulation of γH2AX, marker of DNA damage, in RCE-treated cells. MDA-MB-231 cells were treated with and without increasing concentrations of RCE for 48 h and DNA damage was analyzed, by western blot, by determining the level of γH2AX accumulation using anti- phospho-H2AX (ser 139) antibody. (**E–F**) DNA damage precedes autophagy. (**E**) Time-course measurement of DNA damage and LC3-II accumulation in treated MDA-MB-231 cells. Cells were treated with 400 μg/mL RCE and DNA damage and autophagy induction was examined, as described above, at different time-point (6, 12, 24 and 48 h). (**F**) Effect of autophagy inhibitors on the accumulation of DNA damage. Cells were pretreated with CQ (50 μM) or 3-MA (5 mM) for 1 h before adding RCE (400 and 600 μg/mL) for 48 h. Cells were then harvested and γH2AX level was determined by western blot as described above. The western blots shown are representative of at least three independent experiments.

Increasing evidence underlines the involvement of p38 and ERK1/2 signaling pathways in induction of autophagy^49^,^50^,^51^. Therefore we decided to investigate the status of these two pathways in response to *Rhus coriaria*. Toward this, we treated cells with increasing concentrations of RCE for 48 h and examined the levels of the phosphorylated forms of p38 and ERK1/2. Interestingly, a marked induction of phosphorylation of both p38 and ERK1/2 was noted (Fig. 8A), indicating that these two pathways were activated in response to RCE. To examine the possible link between autophagy and activation of p38 or ERK, a time course study of p38 and ERK1/2 phosphorylation as well as LC3-II accumulation was performed. As shown in Fig. 8B, a sustained increase of phospho-p38 and phospho-ERK1/2 was observed starting 12 h after RCE treatment and lasted for more than 48 h (Fig. 8B). Interestingly, accumulation of the autophagy marker, LC3-II, also started 12 h post-treatment and kept increasing over time (Fig. 8B). Hence, the time course data suggest that activation of p38 and ERK1/2 coincides with the induction of autophagy.

To establish a direct relationship between activation of p38 and ERK signaling and autophagy, the autophagy inhibitors 3-MA and CQ were employed. We found that both 3-MA and CQ (Fig. 8C) completely abrogated the phosphorylation of p38. It is noteworthy to mention that, when used alone, 3-MA led to a reduction of the basal level of phospho-p38, in agreement with previous data in MCF-7 and MDA-MB-231 cells^50^. This result suggests that activated p38 is involved in all the stages of autophagy induction (autophagosome formation and autolysosome maturation). The autophagy inhibitor 3-MA did not lead to significant changes in the level of ERK1/2 phosphorylation; CQ, on the other hand, markedly reduced the level of p-ERK1/2 (Fig. 8C). These results suggest that induction of autophagy by RCE might involve the activation of p38 and ERK in MDA-MB-231 cells.

### *Rhus coriaria* induces DNA damage as an early event that precedes autophagy and senescence in MDA-MB 231 cells

Next, we sought, in the first instance, to investigate whether RCE induces DNA damage in MDA-MB-231 cells. Western blotting analysis revealed a concentration-dependent increase in the levels of phosphorylated H2AX (γH2AX) (Fig. 8D), indicating an accumulation of double strand breaks in treated cells.

In order to assess whether DNA damage is an early event that precedes autophagy and senescence, a time-course measurement of γH2AX (marker of DNA damage) and LC3 II accumulation (marker of autophagy) in cells treated with 400 μg/mL RCE was carried out. We found that activation of γH2AX occurred as early as 6 h post-treatment (Fig. 8E), a time at which no cell death occurred (data not shown). This rule out the possibility that the induction of DNA damage is a consequence of DNA fragmentation. On the other hand, LC3 II significantly accumulated 12 h post-treatment (Fig. 8B), suggesting that autophagy occurs downstream of DNA damage. To further confirm that DNA damage precedes autophagy, MDA-MB-231 cells were first incubated for 1 h with the autophagy inhibitor CQ and then treated with the indicated concentration of RCE. Figure 8F, shows that inhibition of autophagy did not prevent DNA damage in treated cells. Similar results were obtained with 3-MA. Taken together, these results indicate that DNA damage is an earlier event in RCE-treated cells. This damage might then serve as a trigger for downstream responses culminating in autophagy, senescence and cell death. Bases on these findings, we demonstrate that DNA damage is an early response to RCE that might contribute to induce autophagy and senescence in MDA-MB-231 cells.

### *Rhus coriaria* downregulates mutant p53 in MDA-MB-231 cells

**Figure 9:**
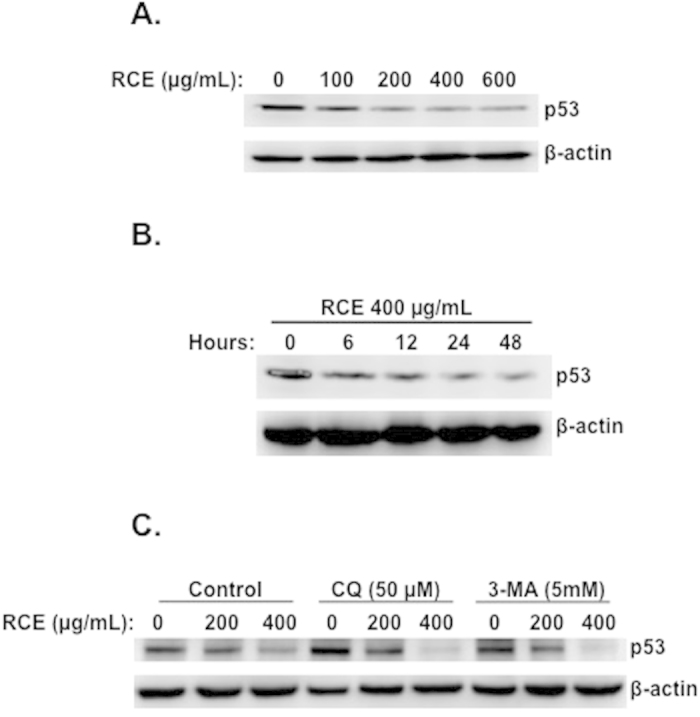
Rhus coriria downregulated the level of p53 in MDA-MB-231 cells. (**A**) Concentration-dependent reduction in the protein level of p53 in RCE-treated cells. MDA-MB-231 cells were treated with and without increasing concentrations of RCE for 48 h and DNA p53 protein level was analyzed by Western blot. (**B**) Time-course measurement of p53 level in treated cells. Cells were treated with 400 μg/mL RCE and p53 level was examined at different time-point (6, 12, 24 and 48 h). (**C**) Effect of autophagy inhibitors on p53 protein level. Cells were pretreated with the indicated concentrations of CQ or 3-MA for 1 h before adding RCE (200 and 400 μg/mL) for 48 h. Cells were then harvested and p53 protein level was determined by western blot as described above. The western blots shown are representative of two independent experiments.

Next, we tested the effect of RCE on the expression of the tumor suppressor p53 in MDA-MB-231 cells. Toward this aim, cells were treated with increasing concentrations of RCE and the protein level of p53 protein was determined. Western blotting analysis revealed a concentrations-dependent decrease of p53 (Fig. 9A). Time-course analysis revealed that the level of p53 decreased as early as 6 h post-treatment (Fig. 9B), suggesting that p53 decrease is an early event that preceded autophagy induction, detected only 12 h post-treatment. To further confirm, that p53 reduction occurred upstream of autophagy, MDA-MB-231 cells were pre-treated with autophagy inhibitors 3-MA and CQ and then treated with RCE at the indicated concentrations for 48 h. Results shown in Fig. 9C, showed that neither 3-MA nor CQ had an effect on p53 reduction, hence demonstrating that p53, like DNA damage, is an early event that might serve as a trigger for the downstream response on MDA-MB-231 cells to RCE treatment. Our results regarding p53 are potentially an important finding because of the role of mutant p53 protein in human cancers. In fact, mutant p53 renders cancer cells more resistant to anticancer drugs, thus, abolishing mutant p53 may offer a promising approach for cancer prevention and therapy.

## Discussion

Increasing evidence indicates that natural compounds from plants can promote cancer cell death through induction of autophagy^23^. In the present study, we investigated the anticancer effect of *Rhus coriaria L*., on three breast cancer cell lines. Our current work demonstrated for the first time that *Rhus coriaria ethanolic extract* (RCE) was able to inhibit the proliferation of three breast cancer cells in a time- and concentration-dependent manner. RCE induced irreversible cell cycle arrest at G1 phase and senescence associated with upregulation of p21, downregulation of cyclin D1, p27, PCNA, p53 and c-myc, hypophosphorylation of the tumor suppressor RB and expression of Senescence-associated-β-Galactosidase (SA-β-Gal) activity. Moreover, we showed that RCE induced autophagy that ultimately led to cell death. Further, we found that RCE-induced autophagy is associated with activation of p38 and ERK.

Stress-induced senescence was shown to occur in tumor cells *in vitro* and *in vivo* through exposure of the cells to cytotoxic agents known to cause DNA damage^9^. Here, we showed that *Rhus coriaria* induced cell cycle arrest and senescence associated with an upregulation of p21, hypophosphorylation of RB and expression of SA-β-Gal. In fact, we demonstrated that p21 expression was robustly increased at concentration of RCE (200 μg/mL) that induced senescence in MDA-MB-231 cells. Based on these data, we propose that induction of senescence in RCE-treated cells is mediated, at least partly, through p21. This conclusion finds support in the following observations. (1) p16 and p27, which are also regarded as key effectors of cellular senescence, were downregulated in cells treated with concentrations of RCE that induced senescence. (2) Senescence was not induced in cells treated with concentrations of RCE (400 and 600 μg/mL) that dramatically reduced the level of p21 protein. Surprisingly, we found that p21 levels decreased when cells were exposed for prolonged time to higher concentrations of RCE (>400 μg/mL). These same concentrations actually precipitated autophagy and cell death in MDA-MB-231 cells. Interestingly, a recent study showed that p21 plays an essential role in determining the type of cell death^52^. Indeed, Fujiwara *et al.* described that stimulation of the cell death signal by C2-ceramide induces caspase-dependent apoptosis in p21^+/+^ MEF cells, while in p21^−/−^ MEFs, C2-ceramide triggered autophagy^52^. Moreover, inhibition of p21 suppresses the apoptotic pathway and turns on the switch that triggers autophagy in p21^+/+^ MEFs, whereas the introduction of p21 increases the sensitivity of p21^−/−^ MEFs to apoptosis. Based on these previous findings, we propose that the decreased levels of p21 observed after 48 h in cells treated with higher concentration of *Rhus coriaria* can serve as a trigger for autophagic cell death in MDA-MB-231 cells. However, the exact mechanism through which high concentration of RCE elicits different effect on p21 levels (upregulated at early time point and downregulated after 48 h) is unclear. A concentration-dependent effect on p21 expression has been described for salinomycin, a polyether antibiotic with anticancer agent activity. In fact, salinomycin was shown to induce cell cycle arrest and senescence at a lower concentration that caused upregulation of p21 in breast cancer cells, while it induced autophagy and cell death at higher concentration that reduced the level of p21 protein^46^,^53^.

Modulating autophagy as a modality for cancer therapy has been receiving increasing attention in the last few years. Autophagy is a highly conserved lysosomal degradation pathway by which misfolded or aggregated proteins, damaged organelles and intracellular pathogens are recycled or eliminated^11^. As such, tight regulation of autophagic processes is integral to cellular homeostasis. Under normal conditions, basal autophagy functions to remove aged and damaged organelles and proteins. Excessive autophagy, however, may, lead to cell death referred to as type II programmed cell death (type II PCD)^12^. Autophagy seems to also play an important role in cancer cell survival or death. It contributes to cytoprotective events that help cancer cells to survive and evade executing cell death programs^54^. In other circumstances, autophagy can stimulate a pro-death signal pathway in cancer cells. Although apoptosis and autophagy can sometimes exert synergetic effects, some reports show that autophagy can be triggered only when apoptosis is suppressed^54^. Here we showed that the *Rhus coriaria*-induced autophagy leads to cell death mainly through type II PCD; apoptosis seems to play a minor role. Hence, in breast cancer cells, autophagy acts as a pro-death mechanism in response to *R. coriaria*. This conclusion is supported by the following results. Firstly, Inhibition of autophagy by 3-MA or CQ, reduced cell death in RCE-treated cells. Secondly, inhibition of autophagy by the two inhibitors did not increase apoptosis in treated cells.

Kinases play an integral role in the inception and execution of autophagy. Several recent studies implicate the mitogen-activated protein kinase p38 and ERK in autophagy and ER stress responses. In fact, ERK was shown to induce autophagy in response to anticancer agents, such as soyasaponins in colon cancer cells^55^, dendropanoxide (DP) in human osteosarcoma cells^56^ and capsaicin in breast cancer cells^50^. In the case of capsaicin, ERK activity was also shown to be required at the step of maturation of autophagic vacuoles and inhibition of ERK resulted in increased LC3-II indicative of degradation blockade^50^. Similarly, Wong and collaborators showed that sustained ERK1/2 activation act as an upstream effector controlling both autophagy and apoptosis in response to high level of intracellular ROS, triggered by a small molecule compound referred a C1, and that its pharmacological inhibition blocked the C1 induced autophagy and apoptosis^57^. Also, inhibition of ERK was associated with a decrease in autophagy and increased cellular sensitivity to tumor necrosis factor-α (TNF) in breast cancer MCF-7 cells^58^. As for p38, there are controversial data as for whether it promotes or inhibits autophagy. Activation of p38 was shown to positively regulate autophagy by induced by bromelain^59^ and capsaicin^50^ in breast cancer or resveratrol in hepatocellular carcinoma cells^60^. Conversely, Thyagarajan *et al.*, showed that induction of autophagy by *Ganoderrma lucidum* in colon cancer cells, requires the inhibition of p38^61^. In the present work, we showed that *R. coriaria* induced a sustained activation of p38 and ERK1/2 in MDA-MB-231 cells. Moreover, the activation of these MAPKs occurred as early as 12 h post-treatment, a time at which autophagy was detected, as revealed by the accumulation of LC3-II. Interestingly, we found that activation of p38 was completely inhibited by 3-MA or CQ, hence suggesting that p38 activation is involved at all stages of autophagy, i.e. (i) formation of autophagosomes and (ii) fusion of autophagosomes with lysosomes leading to the formation of autophagolysosomes. We also found that only CQ reduced significantly the level of ERK1/2 phosphorylation thus, suggesting that ERK1/2 activation intervenes only in the process of the maturation of autophagolysosomes. The question as to whether the p38 and ERK1/2 acts in concert or independently in the process of autophagolysosomes maturation still remains to be eludicated. It is noteworthy to mention that similar mechanistic observations were reported. For example, it was recently shown that p38 is involved in autophagosomes formation, while ERK1/2 modulates the maturation of autophagolysosomes^49^.

There are an increasing number of reports that provides compelling evidence linking cellular senescence and autophagy. Most recent evidence came from the work by Qi *et al.* (2013). They showed that pharmacological inhibition of autophagy by either 3-MA or CQ blocked senescence induced by pseudolaric acid B, a natural compound isolated from *Pseudolarix kaempferi*, in murine aneuploidy fibrosacroma L929 cells^62^. More evidence supporting the notion that autophagy is required for senescence comes from the work of Knizhnik and collaborators. They showed that senescence induced by the chemotherapeutic drug temozolomide (TMZ) was completely abolished by 3-MA in LN-229 glioma cells^63^. Here, we showed the *R. coriaria* induced both senescence and autophagy in breast cancer cells. We also showed that while autophagy was induced starting 12 h post-treatment, senescence however, become detectable only after 48 h treatment and increased over time. Most interesting is that inhibition of autophagy by CQ markedly reduced the ratio of senescent cells. Hence our work adds supplementary evidence supporting the notion that autophagy can be a prerequisite for cellular senescence.

Interestingly, we found that RCE induced a dose-dependent increase in γH2AX, a marker of DNA damage, detected as early as 6 h, a time that preceded autophagy induction and senescence. In fact, autophagy was triggered after 12 h of treatment, while senescence was detectable after 48 h. It appears then from our results that DNA damage is an earlier event in RCE-treated cells. This damage might then serve as a trigger for downstream responses culminating in autophagy, senescence and cell death. In summary to our findings, we believe that the magnitude of DNA damage determines the response of stressed cells. We propose that low concentration of RCE leads to limited DNA damage and cells respond mainly by triggering cell cycle arrest, autophagy followed by cellular senescence as survival mechanism. However exposure of cells to higher concentration of RCE for prolonged time causes overwhelming amount of damage which results in increased autophagy that ultimately lead to cell death.

The tumor suppressor protein, p53, mutated in about 50% of human cancers^64^ was reported to play a key role in cancer cells resistance to certain anticancer drugs and thus considered as a potential cancer-specific target for pharmacologic interventions in human cancers^65^,^66^. Studies have shown that inhibition of mutant p53 by RNA interference sensitizes cancer cell to cell death by chemotherapeutic agents^67^. Wang *et al.* 2011, showed that the naturally occurring isothiocyanates (ITCs) phenetyl isoisothiocyanate (PEITC), derived from watercress plant, and the synthetic ITC, 2,2-di phenetyl isoisothiocyanate selectively deplete mutant, but not the wild-type p53, and induce cell death in many cancer cells, including the MDA-MB-231 breast cancer cells^68^. Moreover, increasing number of evidence incriminate p53 in inhibition of autophagy and its inactivation by knockout, knockdown or pharmacological inhibition induces autophagy in human and mouse cells^69^. Interestingly, here we found that RCE led to marked decrease in the mutant p53 level which occurred upstream of autophagy induction and therefore might contribute, at least partly, to the RCE-mediated autophagy.

In summary, the results of our study demonstrates, for the first time, the potential chemotherapeutic effect of the perennial edible plant, *Rhus coriaria*, on the growth of breast cancer cells *in vitro* through the induction of autophagy and senescence. This study provide preliminary evidences that suggests that *Rhus coriaria*, might be a valuable source of potentially new natural anti-breast cancer compound(s) that act by triggering autophagic cell death. Therefore, this plant deserves more explorations in order to identify the bioactive phytochemical(s) conferring its anti-breast cancer activity.

## Additional Information

**How to cite this article**: El Hasasna, H. *et al.* *Rhus coriaria* induces senescence and autophagic cell death in breast cancer cells through a mechanism involving p38 and ERK1/2 activation. *Sci. Rep.* **5**, 13013; doi: 10.1038/srep13013 (2015).

## Supplementary Material

Supplementary Information
