# Supplementary material for: Rhus coriaria induces senescence and autophagic cell death in breast cancer cells through a mechanism involving p38 and ERK1/2 activation
Source: Sci Rep. 2015 Aug 12;5:13013. doi: 10.1038/srep13013 (PMC4532997; doi:10.1038/srep13013)
Supplement: Supplementary Information [file srep13013-s1.pdf]

Supplementary Figures and Table

Rhus coriaria induces senescence and autophagic cell death in breast cancer cells through a mechanism involving p38 and ERK1/2 activation

Hussain El Hasasna[q1], Khawlah Athamneh[q1], Halima Al Samri[q1], Nushad Karuvantevida[1], Yusra Al Dhaheri[1], Soleiman Hisaindee[2], Gaber Ramadan[1], Nedaa Al Tamimi[1], Synan AbuQamar[1], Ali Eid[3*] and Rabah Iratni[1*]

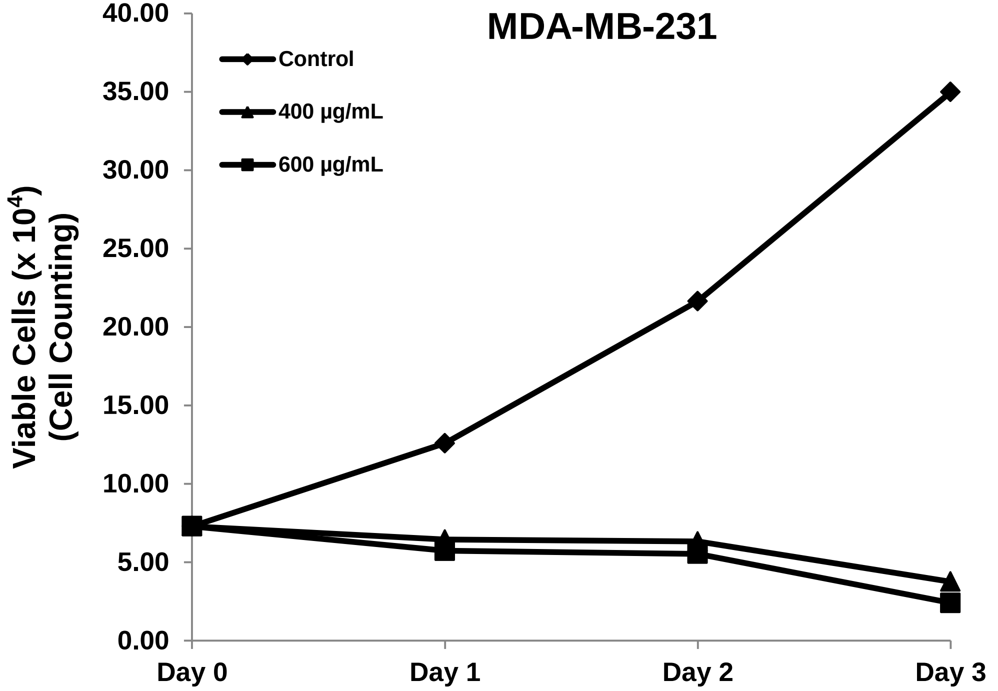

Total Viable Cells (x 10^4)				
	Day 0	Day 1	Day 2	Day 3
Control		12.60 ± 0.37	21.65 ± 2	35.0 ± 1.6
400 µg/mL	7.30 ± 0.39	6.45 ± 0.27	6.33 ± 0.09	3.77 ± 0.45
600 µg/mL		5.74 ± 0.22	5.55 ± 0.5	2.41 ± 0.36

Figure S1. Inhibition of cellular viability by Rhus coriaria. Exponentially growing MDA-MB-231 cells were treated with and without the indicated concentrations of RCE for 1, 2 and 3 days. Viability monitored by counting the number of viable cells as described in Materials and Methods. Data represent the mean of three independent experiments carried out in triplicate.

Supplementary figure 1

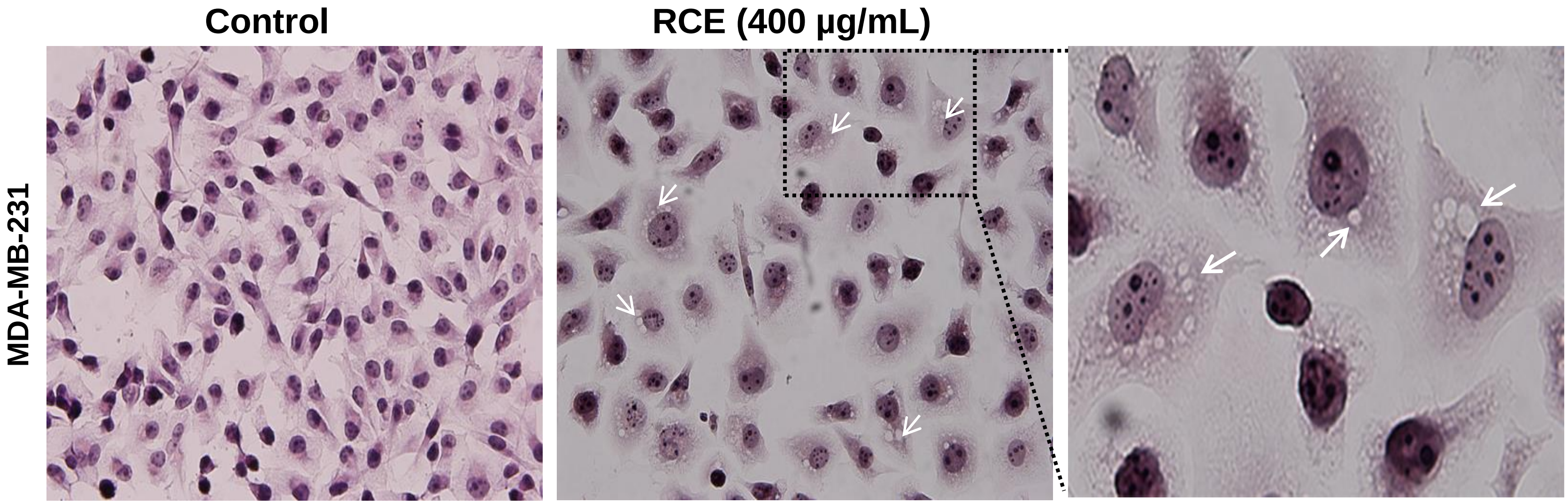

Figure S2. Rhus coriaria induces massive cytoplasmic vacuolation in MDA-MB-231 cells. MDA-MB-231 cells were treated for 48 with RCE (400 µg/mL) and then stained with Eosin-Hemtoxylin as described in Materials and Methods.

Supplementary figure 2

IC$_{50}$ (µg/mL)			
	24 h	48 h	72h
MDA-MB-231	437	305	283
T47D	374	261	229
MCF-7	ND	510	433

Supplementary table 1: IC50 values determined for each cell at the indicated time of treatment.
